# *Burkholderia pseudomallei* Sequence Type 46 Transmission from Asia to Australia

**DOI:** 10.3201/eid3102.241385

**Published:** 2025-02

**Authors:** Ella M. Meumann, Mirjam Kaestli, Jessica R. Webb, Vanessa Rigas, Celeste Woerle, Mark Mayo, Bart J. Currie

**Affiliations:** Territory Pathology, Darwin, Northern Territory, Australia (E.M. Meumann); Menzies School of Health Research and Charles Darwin University, Darwin (E.M. Meumann, M. Kaestli, J.R. Webb, V. Rigas, C. Woerle, M. Mayo, B.J. Currie); Royal Darwin Hospital, Darwin (E.M. Meumann, B.J. Currie); University of Melbourne at The Peter Doherty Institute for Infection and Immunity, Melbourne, Victoria, Australia (J.R. Webb)

**Keywords:** melioidosis, bacteria, respiratory infections, Burkholderia pseudomallei, epidemiology, genomics, Asia, Australia

## Abstract

Melioidosis is caused by the environmental pathogen *Burkholderia pseudomallei*. Among 1,331 patients with melioidosis during 1989–2023 in the Darwin Prospective Melioidosis Study in Australia, we identified 6 locally acquired cases caused by *B. pseudomallei* sequence type 46. Because of global transmission and expansion of endemicity, clinicians should increase awareness of melioidosis.

The environmental bacterium *Burkholderia pseudomallei* causes melioidosis and is globally endemic in tropical and subtropical regions. At the continental level, *B. pseudomallei* populations remain distinct, and phylogeographic analyses suggest an origin in Australia and subsequent dispersal to Asia, Africa, and the Americas (*1*). Intercontinental transmission events are rare but have occurred in association with contaminated products (*2*) and imported animals (*3*).

The Darwin Prospective Melioidosis Study (DPMS) has documented all culture-confirmed melioidosis cases in the Top End of Australia’s Northern Territory since October 1989 (Appendix 1). We previously described emergence of *B. pseudomallei* sequence type (ST) 562 in the Top End; ST562 from Asia is now the most common cause of melioidosis in the Darwin region (*4*–*6*). Another *B. pseudomallei* strain, ST46, likely of origin from Asia, was identified in the DPMS and had caused 6 cases in the region.

Of the 1,374 DPMS melioidosis cases during October 1, 1989–September 30, 2023, multilocus sequence typing was available for 1,331 *B. pseudomallei* isolates, 6 of which were ST46 (Table). The ST46 occurrences were during the wet season (November–April) during 2013–2023. Of the 6 case-patients, 5 resided in the rural area (30–35 km south of Darwin) and 1 in the urban area; none reported recent overseas travel. Of the 5 persons from the rural area, 4 reported recent gardening activities. The person from the urban area visited the rural area but did not report any specific environmental exposure. All 6 persons sought treatment for community-acquired pneumonia, 4 had *B. pseudomallei* isolated from blood cultures, and 2 had septic shock. All 6 patients survived.

**Table:** Epidemiology and clinical features of *Burkholderia pseudomallei* cases identified in Australia as part of a study of intercontinental transmission of *B. pseudomallei* sequence type 46*

DPMS ID	Age, y/sex	Exposure risk	Underlying conditions	Location, year	Clinical manifestations
861	57/M	Landscape grading	Hazardous alcohol consumption, COPD	Rural area of Darwin, 2013	Acute pneumonia
1100	62/M	Lawn mowing	Diabetes, hazardous alcohol consumption	Rural area of Darwin, 2018	Acute pneumonia with septic shock
1102	43/F	Lawn mowing	Diabetes, previous lymphoma	Rural area of Darwin, 2018	Acute pneumonia with septic shock
1266	78/M	Repotting plants	Myelofibrosis, prostate cancer	Rural area of Darwin, 2022	Acute pneumonia
1329	41/M	Camping	Diabetes, hazardous alcohol consumption	Rural area of Darwin, 2022	Chronic pneumonia
1401	31/M	No identified exposure	No underlying conditions	Urban area of Darwin, 2023	Acute pneumonia with secondary soft tissue abscess left leg

*COPD, chronic obstructive pulmonary disease; DPMS ID, Darwin Prospective Melioidosis Study identification number.

Isolation from the environment is crucial for confirming local establishment of *B. pseudomallei* ST46. Despite extensive sampling in the Darwin region as part of previous studies (*7*,*8*), we did not find *B. pseudomallei* ST46 in the environment. Six *B. pseudomallei* ST46 genomes in the context of 41 publicly available global ST46 genomes, 128 genomes from other Australia cases in the DPMS, and 149 publicly availably international genomes underwent phylogenetic analysis (Appendix 2 Table). The 6 ST46 genomes from our study were closely related to ST46 genomes from Asia and, along with ST562, were within the Asian clade of the global phylogeny (Figure, panel A). The 6 ST46 genomes from Australia were closely related, separated by only 3–9 single-nucleotide polymorphisms (SNPs). Among all 47 ST46 genomes, median pairwise distance was 100 SNPs (maximum 176 SNPs).

**Figure:**
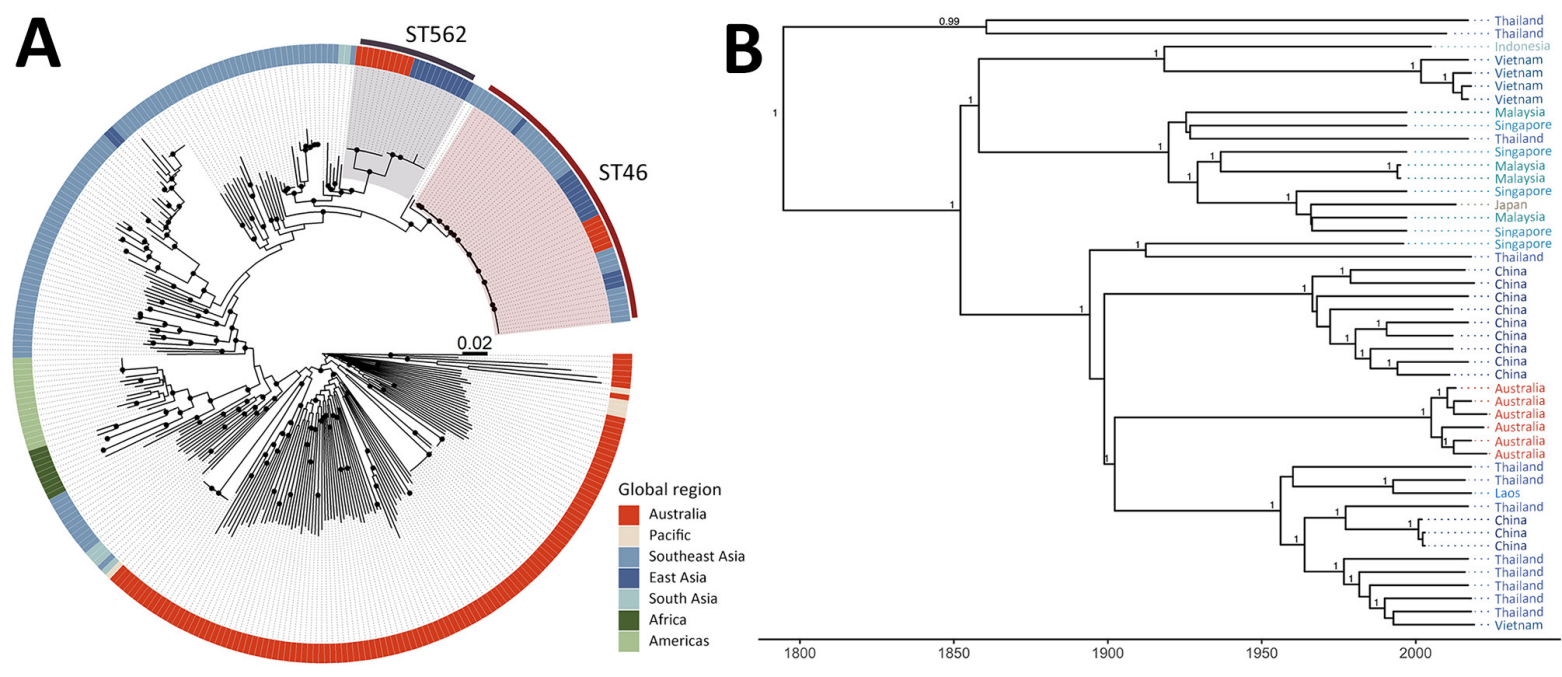
Phylogenies of *Burkholderia pseudomallei* ST46 shown in an investigation of *B. pseudomallei* ST46 transmission from Asia to Australia. A) Midpoint-rooted maximum-likelihood global phylogeny; B) maximum clade credibility tree. Trees include genomes collected as part of the Darwin Prospective Melioidosis Study in Darwin, Northern Territory, Australia, and others available in public sources (Appendix 1 Table). Black circles indicate nodes with an approximate likelihood ratio >95 and ultrafast bootstrap >95. Labels indicate nodes with posterior support >0.8. ST, sequence type.

We generated an ST46-only time-calibrated tree (Figure, panel B; Appendix 1). The 6 ST46 genomes from Australia formed a single clade, and the most recent common ancestor was predicted to have occurred in 2004 (95% highest posterior density 1994–2012). Genomes from across Asia, including southern China, countries surrounding the Mekong, the Malay peninsula, and Indonesia, were interspersed within the tree, and the most recent common ancestor for the whole phylogeny was predicted to have occurred in 1775 (95% highest posterior density 1598–1897). In PubMLST (https://pubmlst.org/organisms/burkholderia-pseudomallei), ST46 is the most common ST found in Asia.

The source of *B. pseudomallei* ST46 introduction to northern Australia is unclear. Within the ST46 phylogeny, the 6 genomes from Australia sit within a clade comprising genomes from Hainan and Guangdong Provinces in China (*9*) (Appendixes 1, 2) and several provinces in northeast Thailand (Appendixes 1, 2). Of note, ST562 is also reported from Hainan (*6*–*8*) and Guangdong (*9*) Provinces.

Although previous studies in northern Australia showed that most STs are highly geographically restricted (*7*,*8*), phylogeographic analyses of *B. pseudomallei* in Asia showed those STs are more geographically dispersed, and STs often span multiple countries (*1*) (Appendix 1). That dispersal may occur through large river systems such as the Mekong River, airborne transmission in association with strong winds, transport through historic and current trade routes, extensive agriculture (e.g., rice paddies), and high population density (*1*) (Appendixes 1, 2). Although *B. pseudomallei* genomes from Thailand are distributed throughout most clades in the ST46 phylogeny, suggesting Thailand (or the Mekong region) as a possible ST46 transmission source, because of sampling limitations, determination of the origin of ST46 is not possible.

Arrival mode of ST46 in northern Australia remains uncertain and is difficult to ascertain but might relate to imported animals, plants, other products, or migratory birds. The Darwin rural area is experiencing strong growth and is an agricultural hub comprising fruit farms and residential rural blocks with animals. The area has several lagoons and wetlands nearby with abundant birdlife. Importation through 1 of those routes is more likely than a severe weather event because the Darwin rural area has an inland location, and *B. pseudomallei* is likely to be inactivated by ultraviolet light on such a long journey (*10*).

In summary, global epidemiology of melioidosis is changing with increasing globalization, environmental disturbance associated with construction and urbanization, and severe weather events associated with climate change. Genomics is crucial for understanding that dynamic situation, including identifying long-range transmission events and differentiating those from previously unrecognized endemicity. Because of the global spread and potential for transmission, clinicians should increase their awareness of melioidosis and its manifestations.

Appendix 1 Additional methods for investigation of *Burkholderia pseudomallei* sequence type 46 transmission from Asia to Australia.

Appendix 2 Accession numbers and locations for genomes for investigation of *Burkholderia pseudomallei* sequence type 46 transmission from Asia to Australia.
