# Supplementary material for: Burkholderia pseudomallei Sequence Type 46 Transmission from Asia to Australia
Source: Emerg Infect Dis. 2025 Feb;31(2):394–7. doi: 10.3201/eid3102.241385 (PMC11845124; doi:10.3201/eid3102.241385)
Supplement: Appendix 1 — Additional methods for investigation of Burkholderia pseudomallei sequence type 46 transmission from Asia to Australia. [file 24-1385-Techapp-s1.pdf]

Article DOI: https://doi.org/10.3201/eid3102.241385

EID cannot ensure accessibility for supplementary materials supplied by authors. Readers who have difficulty accessing supplementary content should contact the authors for assistance.

Burkholderia pseudomallei Sequence Type 46 Transmission from Asia to Australia

Appendix

Supplementary Methods

This study was approved by the Human Research Ethics Committee of the Northern Territory Government and Menzies School of Health Research. The data collected as part of the Darwin Prospective Melioidosis Study included patient demographics, underlying conditions, clinical presentations, and outcomes of patients with melioidosis (*1*). *B. pseudomallei* isolates for each case underwent whole genome sequencing by using an Illumina (short-read) platform and in silico multilocus sequence typing (MLST; https://pubmlst.org/organisms/burkholderia-pseudomallei).

Phylogenetic analysis was conducted on the 6 DPMS *B. pseudomallei* ST46 genomes in the context of 41 publicly available global ST46 genomes (all from Asia) (*2–4*), 128 genomes from other Australian cases in the DPMS (*1*), and 149 international genomes (*5–8*) (Appendix 1, Table). Sequences were aligned to the K96243 reference genome (accession no. GCA_000959285.1) by using Snippy v4.6.0 (https://github.com/tseemann/snippy) and thresholds of ≥10× coverage and ≥90% prevalence to call variants. The core single nucleotide polymorphism (SNP) alignment was used for phylogenetic analysis in IQ-TREE v2.2.0.3 (*9*) by using a generalized time-reversible (GTR) model, 4 gamma categories, 1,000 ultrafast bootstrap replicates, and 1,000 approximate likelihood ratio replicates.

To further investigate the relationship between the Australian and Asian ST46 isolates, the ST46 genomes were aligned to the closed ST46 *B. pseudomallei* Tokushima genome (accession no. GCA_030297295.1) by using Snippy as described above. Regions of recombination were predicted with ClonalFrameML v1.2 (*10*) and were masked from the alignment. Temporal analysis was conducted with BEAST 2 (*11*). The temporal analysis

included 953 core SNPs, with constant sites included in the .xml file. HKY and GTR substitution models and strict and relaxed clock models were tested; each of those analyses converged with no major differences in estimates of the clock rate or tree height. The final analysis was done with a GTR model with 4 gamma categories, a relaxed clock model with log-normal distribution of rates (with clock rate prior set as a log-normal distribution with mean 1E^–6 and 95% highest posterior density ranging from 3.95E^–8 to 5.31E^–6), and a coalescent constant population model. Ten replicates were run, each with 800 million iterations, and with sampling from the posterior every 80,000 iterations; examination of the trace files revealed that those all converged and resulted in similar estimates of the tree height and clock rate. The logs and trees were combined by using LogCombiner with 15% burn-in and resampling of every 800,000th iteration. Effective sample sizes for all parameters were >700. A maximum clade credibility tree was calculated by using TreeAnnotator. A replicate sampling from the prior only with no sequence data did not converge, and 10 replicates with randomized tip dates did converge but had wildly varying estimates of the tree height and clock rate with wide 95% HPD estimates; those findings provided support that the priors were not driving the results of our analysis.

References

1. Currie BJ, Mayo M, Ward LM, Kaestli M, Meumann EM, Webb JR, et al. The Darwin Prospective Melioidosis Study: a 30-year prospective, observational investigation. Lancet Infect Dis. 2021;21:1737–46. PubMed https://doi.org/10.1016/S1473-3099(21)00022-0

2. Nandi T, Holden MT, Didelot X, Mehershahi K, Boddey JA, Beacham I, et al. *Burkholderia pseudomallei* sequencing identifies genomic clades with distinct recombination, accessory, and epigenetic profiles. Genome Res. 2015;25:608. PubMed https://doi.org/10.1101/gr.177543.114

3. Zheng H, Qin J, Chen H, Hu H, Zhang X, Yang C, et al. Genetic diversity and transmission patterns of *Burkholderia pseudomallei* on Hainan Island, China, revealed by a population genomics analysis. Microb Genom. 2021;7:000659. PubMed https://doi.org/10.1099/mgen.0.000659

4. Seng R, Chomkatekaew C, Tandhavanant S, Saiprom N, Phunpang R, Thaipadungpanit J, et al. Genetic diversity, determinants, and dissemination of *Burkholderia pseudomallei* lineages implicated in melioidosis in northeast Thailand. Nat Commun. 2024;15:5699. PubMed https://doi.org/10.1038/s41467-024-50067-9

5. Chewapreecha C, Holden MT, Vehkala M, Välimäki N, Yang Z, Harris SR, et al. Global and regional dissemination and evolution of *Burkholderia pseudomallei.* Nat Microbiol. 2017;2:16263. PubMed https://doi.org/10.1038/nmicrobiol.2016.263

6. Cossaboom CM, Marinova-Petkova A, Strysko J, Rodriguez G, Maness T, Ocampo J, et al. Melioidosis in a resident of Texas with no recent travel history, United States. Emerg Infect Dis. 2020;26:1295–9. PubMed https://doi.org/10.3201/eid2606.190975

7. Gee JE, Bower WA, Kunkel A, Petras J, Gettings J, Bye M, et al. Multistate outbreak of melioidosis associated with imported aromatherapy spray. N Engl J Med. 2022;386:861–8. PubMed https://doi.org/10.1056/NEJMoa2116130

8. Petras JK, Elrod MG, Ty MC, Dawson P, O'Laughlin K, Gee JE, et al. Locally acquired melioidosis linked to environment: Mississippi, 2020–2023. N Engl J Med. 2023;389:2355–62. PubMed https://doi.org/10.1056/NEJMoa2306448

9. Nguyen LT, Schmidt HA, von Haeseler A, Minh BQ. IQ-TREE: a fast and effective stochastic algorithm for estimating maximum-likelihood phylogenies. Mol Biol Evol. 2015;32:268–74. PubMed https://doi.org/10.1093/molbev/msu300

10. Didelot X, Wilson DJ. ClonalFrameML: efficient inference of recombination in whole bacterial genomes. PLOS Comput Biol. 2015;11:e1004041. PubMed https://doi.org/10.1371/journal.pcbi.1004041

11. Bouckaert R, Heled J, Kühnert D, Vaughan T, Wu CH, Xie D, et al. BEAST 2: a software platform for Bayesian evolutionary analysis. PLOS Comput Biol. 2014;10:e1003537. PubMed https://doi.org/10.1371/journal.pcbi.1003537
